# Limitations of *Ab Initio* Predictions of Peptide Binding to MHC Class II Molecules

**DOI:** 10.1371/journal.pone.0009272

**Published:** 2010-02-17

**Authors:** Hao Zhang, Peng Wang, Nikitas Papangelopoulos, Ying Xu, Alessandro Sette, Philip E. Bourne, Ole Lund, Julia Ponomarenko, Morten Nielsen, Bjoern Peters

**Affiliations:** 1 Center for Biological Sequence Analysis, Department for Systems Biology, Technical University of Denmark, Lyngby, Denmark; 2 La Jolla Institute for Allergy and Immunology, La Jolla, California, United States of America; 3 San Diego Supercomputer Center and Skaggs School of Pharmacy & Pharmaceutical Sciences, University of California San Diego, San Diego, California, United States of America; 4 Department of Biochemistry and Molecular Biology, Institute of Bioinformatics, University of Georgia, Athens, Georgia, United States of America; Leeds Institute of Molecular Medicine, United Kingdom

## Abstract

Successful predictions of peptide MHC binding typically require a large set of binding data for the specific MHC molecule that is examined. Structure based prediction methods promise to circumvent this requirement by evaluating the physical contacts a peptide can make with an MHC molecule based on the highly conserved 3D structure of peptide:MHC complexes. While several such methods have been described before, most are not publicly available and have not been independently tested for their performance. We here implemented and evaluated three prediction methods for MHC class II molecules: statistical potentials derived from the analysis of known protein structures; energetic evaluation of different peptide snapshots in a molecular dynamics simulation; and direct analysis of contacts made in known 3D structures of peptide:MHC complexes. These methods are *ab initio* in that they require structural data of the MHC molecule examined, but no specific peptide:MHC binding data. Moreover, these methods retain the ability to make predictions in a sufficiently short time scale to be useful in a real world application, such as screening a whole proteome for candidate binding peptides. A rigorous evaluation of each methods prediction performance showed that these are significantly better than random, but still substantially lower than the best performing sequence based class II prediction methods available. While the approaches presented here were developed independently, we have chosen to present our results together in order to support the notion that generating structure based predictions of peptide:MHC binding without using binding data is unlikely to give satisfactory results.

## Introduction

A common bioinformatics application in immunology is the prediction of peptide binding to MHC molecules [1]. Most such binding predictions are based on machine learning algorithms, which aim to generalize experimental binding data to define a binding sequence pattern for a given MHC molecule. The quality of such predictions is therefore highly dependent on the amount of experimental training data available [2]. Moreover, there are thousands of different MHC alleles in the human population and binding data is only available for a small subset of alleles. Therefore, it is desirable to develop binding prediction methods that do not rely on the availability of peptide:MHC binding data.

A promising approach that does not require binding data is to use 3D structures of peptide:MHC complexes. Different MHC alleles have high sequence homology, and all solved MHC structures have a highly conserved fold, which opens the possibility to use homology modeling for those MHC alleles for which no 3D structure has been solved explicitly. Moreover, a structure-based predictive understanding of peptide:MHC binding provides a physical explanation for the nature of the binding interactions, while purely peptide sequence based learning methods merely provide a description of the sequence characteristics of preferred MHC-binding ligands. Throughout this manuscript, we refer to prediction approaches that use structural information but not peptide:MHC binding data as ‘*ab initio*’ approaches.

Several approaches have been published that predict peptide binding to MHC molecules utilizing known 3D structures. Threading-based approaches have been used to align peptides to know peptide:MHC structures and binders are selected using statistical pairwise potentials [3], [4]. Bordner and Abagyan utilized a Biased-Probability Monte Carlo docking protocol to predict peptide:MHC binding [5]. Bui et al [6] developed a *de novo* approach to sample conformations of peptide:MHC backbone and side chains with consideration of explicit water molecules whereas Schafroth and Floudas utilized implicit solvation for their approach [7]. In a separate study, Fagerberg et al [8] utilized molecular dynamic and simulated annealing to sample the conformational space and predict binding of peptides to MHC class I molecules. A similar approach was taken by Davis et al. [9] for the prediction of MHC class II peptide binding. In a recent paper Singh et al. applied threading guided by a structure derived contact potential to predict binding of peptides to MHC class I molecules [10]. Structure information has also been coupled with experimental data to predict peptide:MHC binding via quantitative structure-affinity relationship methods [11]. Evaluation of those methods was typically done using existing structures or a small dataset of known binders and none of them currently provides a public web server. Finally, so-called pan-specific MHC binding predictors have been developed in recent years integrating structural information with experimental peptide binding data allowing for generalization of binding predictions to MHC molecules characterized with few or even no peptide binding data [12], [13], [14], [15], [16], [17] [18].

Here, we present three *ab initio* structure-based approaches for predicting peptide binding to MHC class II molecules. The approaches are based on 1) statistical potentials derived from the analysis of known protein structures, 2) energetic evaluation of different peptide snapshots in a molecular dynamics simulation, and 3) direct analysis of contacts made in known 3D structures of peptide:MHC complexes. Their prediction performance was evaluated rigorously on a large dataset of 3,882 peptide binding affinities to HLA-DRB1*0101. The implementation and evaluation of the three approaches were initially pursued independently by subgroups of the authors at different institutions, but led to overall comparable results: they make significantly better than random discriminations of binders from non-binders, but fail to reach the prediction quality necessary for practical applications.

## Results

This section is separated into two parts: In the first part, results are reported that were generated during the derivation of each of the three structure-based prediction methods, starting with the statistical pair potential-based method, followed by the molecular dynamics simulation and the contact map-based method. In the second part, the derived predictions are applied to a common benchmark set, namely a large set of HLA-DRB1*0101 binding data.

### Derivation of Statistical Pair Potential Predictions

#### The effect of the center of interaction

**Figure 1:**
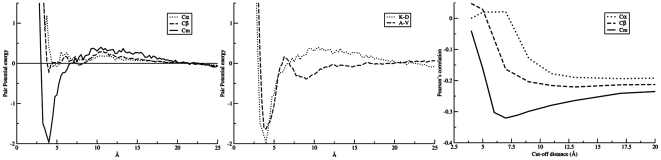
Pairwise potential function. (Left) Pair potential score as a function of interaction distance for K-D based on definition of C_α_, C_β_, and C_m_, respectively. (Middle) Interaction score as a function of C_m_ distance between two hydrophobic amino acids, A-V, and two negatively charged amino acids, L-A, respectively. (Right) Predictive performance as a function of the interactions distance cutoff for three types of interaction centers. The predictive performance is estimated in terms of the Pearsons correlation for the 1173 peptide data in the training data set.

Different schemes of representing the centre of interaction were used in this study: Cα, representing a residue by the alpha carbon; Cβ, by the beta carbon; and Cm, a virtual atom denoting the centre of mass on the sidechain atoms (see material and method). The effect of different implementations of centre of interaction on the pair potential for the K-D residue pair is illustrated in Figure 1. It was expected that the positively charged side-chain of lysine (K) attracts the negatively charged carboxylate group in the side-chain of aspartic acid (D) at a short favorable distance. For the statistical potentials, such a binding energy minimum can be seen clearly for Cm, whereas it was not pronounced for Cα and Cβ. This suggested that the Cm representation was the most suitable for our study. Two additional potentials are shown in Figure 1 for the interaction between pairs of hydrophobic and negatively charged amino acids, respectively. These plots further demonstrate that the calculated potentials for Cm interactions agree with what is expected physico-chemically.

#### The optimal scoring function

The distance cutoff in the scoring function defining which pairwise interactions are taken into account when estimating the binding affinity was estimated based on a benchmark set of MHC class I binding data described in the methods section. For each of the three types of interactions centers, the predictive performance for the training set in terms of the Pearson Correlation Coefficient (PCC) was reported as a function of the cutoff distance used in the scoring function. The results of this calculation are shown in Figure 1. It clearly demonstrates that the predictive performance depends strongly on the type of interaction center, and that the optimal scoring function is found when using the Cm interaction centers with a distance cutoff for interactions at 7.5 Å.

To confirm the validity of the potential scoring function and the optimized potential parameters, we tested its performance on the separate benchmark set of 36,210 peptides that covers 41 MHC class I alleles. In this experiment the built-in Modeller energy was found to correlate poorly with the peptide:MHC binding affinity and had an average PCC of 0.04, whereas the statistical potential for Cα, Cβ and Cm reached an average PCC of 0.11, 0.13 and 0.21, respectively. The pair-potential binding prediction method shows large variations in predictive performance for different MHC molecules. The method performs best for alleles with hydrophobic amino acid preference at the primary anchor positions (A2, and A24 supertype alleles) and worse for alleles with charged amino acid preference at the primary anchor positions (A3, and B44 supertype alleles). For details on this experiment see Table S1. These results confirmed that the potential function based on Cm interaction centers performed better than both Cα and Cβ, and we shall use this potential function with a distance cutoff of 7.5 Å in the subsequent evaluation on the MHC class II benchmark data set described below. Note, that the sequence-based method, NetMHCpan-1.0, evaluated using a leave-one-allele-out approach on the same data set, achieved a performance of 0.674.

### Derivation of Molecular Dynamics-Based Predictions

#### Structures from MD simulation

**Figure 2:**
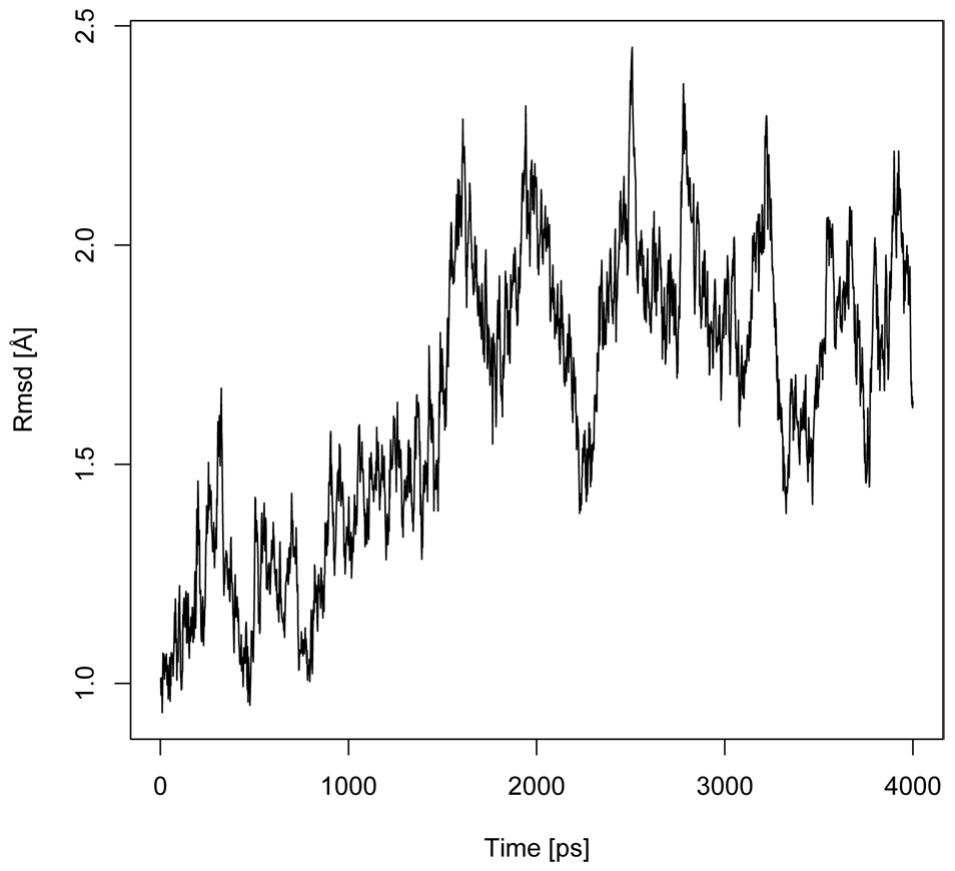
Evolution of RMSD (Å) of the 2G9H protein backbone over 4 ns of MD simulation. Structures of the MD simulation snapshots are aligned to the initial 2G9H structure and their backbone RMSDs (y-axis) are plotted against the time when the snapshots are taken (x-axis). The graph indicates that the structure reaches equilibrium around 1.6 ns into the MD simulation and remains stable through the end of the 4 ns simulation.

For the peptide:MHC class II complex, an MD simulation was carried out for 4 ns. The time-series of the root-mean-square-deviation (RMSD) of backbone atoms from the initial PDB structures is shown in Figure 2. For the 4 ns trajectory, the protein complex has an average RMSD of 1.62 Å with a standard deviation of 0.33 Å. At around 1.6 ns into the simulation, the RMSD of the peptide:MHC complex stabilized around 1.83 Å with a peak value of 2.42 Å suggesting the system has reached equilibrium. In addition, the small RMSD value suggested that the peptide:MHC complex structure is very stable.

#### Binding free energy calculations and its application to binding prediction

From the trajectory of the MD simulation, it is possible to calculate the absolute binding free energy of individual amino acids. This can be done either by binding free energy decomposition [19], [20] or via computational alanine scanning [21]. Previous studies have shown that binding free energy decomposition generally provided more accurate results than computational alanine scanning [22]. However, computational alanine scanning is more suitable for our task as binding free energy decomposition requires MD simulation for large numbers of mutated structures which are prohibitively time consuming. To estimate the binding free energy contributions of all twenty amino acids at each of the nine peptide positions that interact with the MHC class II molecule, we conducted extensive in silico mutations (see Materials and Methods for details). This computational alanine scanning-like approach probed all one hundred eighty (20×9) combinations of amino acids (20) and the peptide core positions (9). Those probing structures generated from the computational alanine scanning like approach were first energy minimized then subjected to binding free energy calculations using the MM-PBSA approach. This process was repeated for 100 snapshots taken from the MD simulation trajectory and the average results were reported as estimates for binding free energy of each amino acid at different core positions.

The calculated absolute binding free energies are displayed in Table 1 in a matrix format. Previous studies have suggest that for HLA-DRB1*0101, binding pocket number one has a strong preference for amino acids with a large neutrally charged side chain [23]. Our calculated binding free energies are consistent with this observation since residues like phenylalanine or tryptophan have the most favorable energies. The structure of the peptide:MHC complexes also suggest that epitope residues at pocket number five will not contribute much to the binding as the side chains protrude away from the MHC class II molecule [24]. Our calculated results are consistent with this finding, as the calculated values for pocket number five deviate less from zero than at other positions.

**Table 1:** Binding free energy contribution of each amino acid at different epitope core locations.

aa\pos	1	2	3	4	5	6	7	8	9
ALA	0.00	0.00	0.00	0.00	0.00	0.00	0.00	0.00	0.00
ARG	1.88	4.71	8.55	9.80	2.67	6.45	10.54	3.95	13.93
ASN	1.47	0.68	1.94	0.85	1.39	1.17	0.64	1.53	3.18
ASP	−14.60	−2.82	−8.95	−5.53	−2.69	−11.09	−9.06	−6.01	−15.69
CYS	2.90	3.47	2.11	1.90	1.05	2.62	3.41	0.98	4.60
GLU	−14.82	−1.89	−8.90	−1.14	−3.13	−11.84	−11.20	−4.60	−15.98
GLN	3.87	2.86	4.08	8.34	1.04	3.75	6.21	1.39	6.12
GLY	−0.86	−1.48	−0.52	−1.74	0.31	−0.81	−2.51	0.00	−1.66
HIS	5.38	1.00	2.31	5.50	1.82	2.86	5.62	1.05	3.72
ILE	4.88	2.38	4.09	2.84	1.84	3.79	3.30	1.11	4.31
LEU	6.59	2.25	3.55	5.22	0.24	2.44	5.43	1.22	6.98
LYS	−5.99	1.59	10.68	5.20	3.35	3.79	3.61	2.40	2.51
MET	8.22	4.41	7.42	8.29	0.02	6.32	5.69	1.45	9.45
PHE	12.14	2.16	6.12	6.45	2.60	3.37	6.19	1.37	8.66
PRO	0.60	−3.51	0.93	−2.79	2.32	0.01	1.98	−2.71	0.60
SER	−1.25	0.36	−0.05	−1.52	0.85	1.03	1.64	0.19	1.16
THR	0.57	1.23	−0.69	0.23	0.65	3.66	0.80	0.20	0.42
TRP	13.49	2.02	6.32	−5.37	3.42	5.03	10.29	1.43	7.35
TYR	12.20	2.06	5.44	3.37	3.20	4.02	7.05	1.22	12.80
VAL	4.48	1.42	0.95	2.15	0.58	2.81	2.46	0.42	2.67

Each row is an amino acid and the columns refer to pocket one to nine of the MHC class II epitope-binding groove. Each value is the difference of binding free energy in comparison with alanine in units of kcal/mol. Positive values indicate residues favorable for binding.

#### Flexibility of epitope and MHC residues during MD simulation

**Figure 3:**
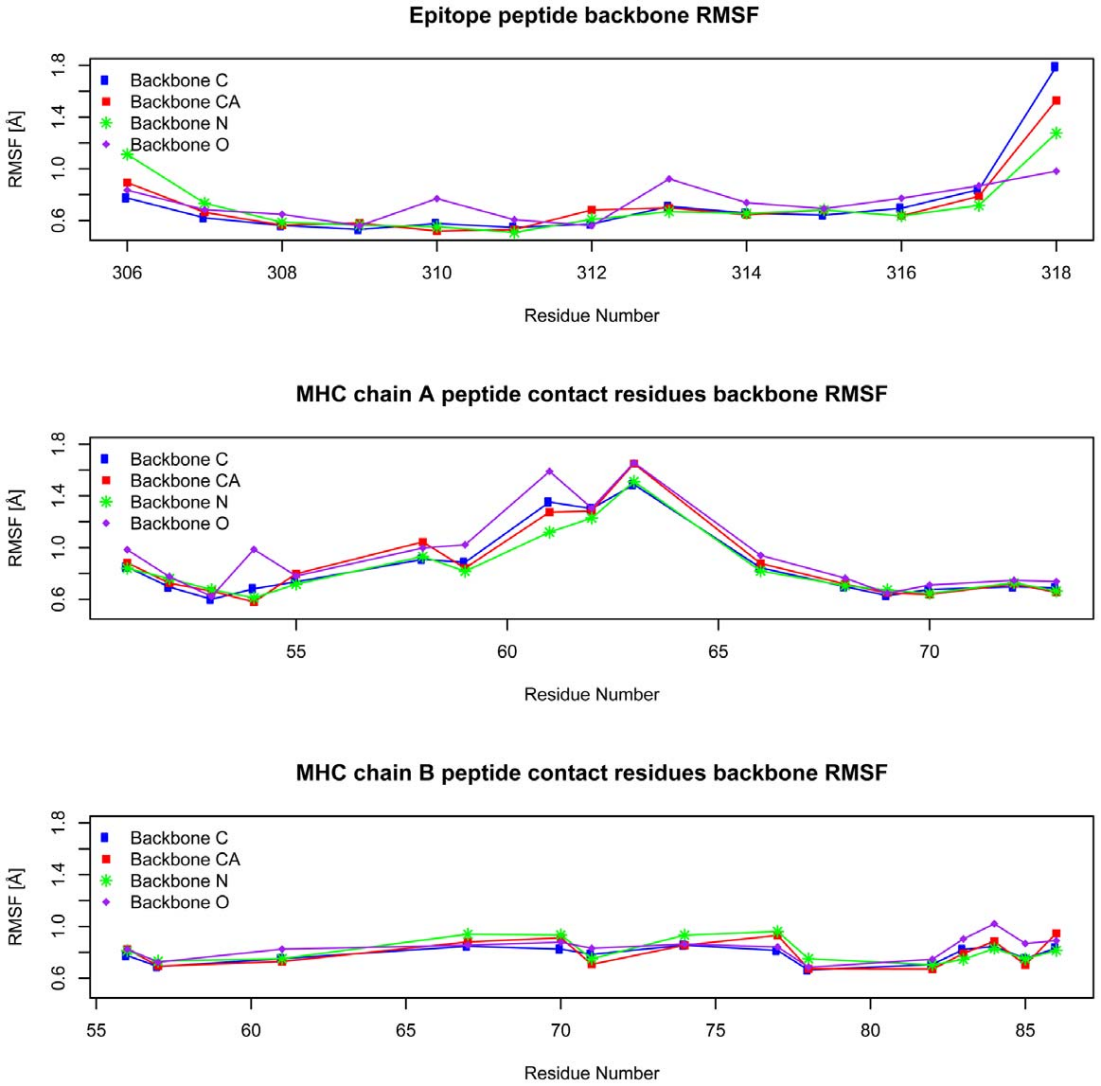
Backbone RMSF (Å) of the epitope peptide and MHC residues contacting epitope over the last 2 ns of MD simulation. RMSFs of the backbone atoms (CA, C, N and O) are plotted against the residue numbers (x-axis). For the epitope peptide, residue 308 is located in pocket 1 of the MHC binding groove and residue 316 is located in binding pocket 9. The MHC residues contact epitope peptide in a linear fashion. For chain A of MHC molecule, the lower numbered MHC residues contact lower numbered peptide residues and higher numbered residues contact higher numbered peptide residues. For chain B of MHC molecule, the contacts are in reverse order in that the higher numbered MHC residues contact lower numbered peptide residues and lower numbered MHC residues contact higher numbered peptide residues.

Dynamic changes of protein structures play important roles in biological processes such as kinase activation and HIV entry into host cell [25], [26]. Utilizing the MD simulation data, we examined the flexibility of the MHC molecule and the peptide epitope by calculating root mean square fluctuation (RMSF) of the peptide backbone atoms and the backbone atoms of MHC residues interacting with peptide (within 5 Å of the peptide). The resulting RMSFs are displayed in Figure 3. The 9mer core residues of epitope peptide (residue 308 to 316) are very stable as their backbone atoms showed very small RMSFs. While the +1 and −1 residues (residue 307 and residue 317) shared similar RMSFs with the core residues, the +2 and −2 residues (residue 306 and residue 318) showed significantly increased RMSFs. This suggested that residues beyond +1 and −1 positions are unlikely to contribute much to peptide:MHC binding as their excessive motions will prevent stable interactions. While peptide interacting residues in chain B of the MHC molecule demonstrated remarkable stability, chain A residues located in the middle portion of the peptide interacting helix showed increased mobility. This suggested that the center region of the peptide binding groove has increased flexibility. This flexibility may help in the incorporation of peptides with diverse residues at the center and provide increased flexibility for T-cell receptor interaction.

### Derivation of Contact-Map Based Predictions

#### Types of atom contacts considered

First, we determined which contacts should be considered in calculating the position specific scoring matrices (PSSM). Four schemas for counting atomic interactions were considered: (1) interactions at a distance of 4 Å; (2) hydrogen bonds alone; (3) van der Waals interactions and hydrogen bonds; and (4) hydrogen bonds together with van der Waals and hydrophobic interactions. Each interacting atom pair was counted once, independent of how many different interactions it participated in. The number of contacts for each amino acid residue was defined as the number of atom-atom interactions in which its atoms were involved while interacting with MHC. To select the schema, we used a benchmark set of MHC class II alleles other than HLA-DRB1*0101 (Table S2). The PSSMs were generated for each MHC class II allele using 3D structures of the peptide:MHC complexes and Eqs. (3.1–3.3). The models based on hydrogen bonds, van der Waals, and hydrophobic interactions gave the best AUC values, while the schema taking into account only hydrogen bonds gave the worst prediction (Table S3).

#### Derivation of contact map PSSM for HLA-DRB1*0101

For each peptide core residue, we calculated the number of contacts in all six complexes with HLA-DR1*0101, taking into account hydrogen bonds, van der Waals, and hydrophobic interactions (Table S4). The values in the table correspond to Q(i,s) in Eq. (3.2), that is the number of times the amino acid of type s is found at position i of the peptide core. Using Eqs. (3.1–3.3), the PSSM for DRB1*0101 was calculated (Table 2). As for the absolute binding free energies calculated with the molecular dynamics method (Table 1), the contact-based PSSM values (Table 2) are consistent with the observation that the HLA-DRB1*0101 binding pocket number one has a preference for hydrophobic amino acids [24]. The contact-based PSSM values are also in agreement with the experimentally measured preferences for the HLA-DRB1*0101 binding pocket number four [23], which mostly favors leucine and methionine and disfavors aspartic acid, lysine, tryptophane, and arginine.

**Table 2:** PSSM for the DRB1*0101 generated by the contact-based method.

aa\pos	1	2	3	4	5	6	7	8	9
ALA	−3.00	−3.00	−3.00	−3.00	0.99	1.50	1.33	−3.00	−3.00
ARG	−3.00	1.81	−3.00	−3.00	2.07	−3.00	−3.00	−3.00	−3.00
ASN	−3.00	−3.00	−3.00	−3.00	1.24	−3.00	−3.00	−3.00	−3.00
ASP	−3.00	−3.00	0.92	−3.00	−3.00	−3.00	−3.00	−3.00	−3.00
CYS	−3.00	−3.00	−3.00	−3.00	−3.00	−3.00	−3.00	−3.00	−3.00
GLU	−3.00	−3.00	−3.00	0.85	−3.00	−3.00	−3.00	−3.00	−3.00
GLN	−3.00	−3.00	−3.00	1.72	0.77	−3.00	−3.00	−3.00	1.23
GLY	−3.00	0.89	0.29	−3.00	−3.00	1.02	−3.00	−3.00	0.32
HIS	−3.00	−3.00	−3.00	−3.00	−3.00	−3.00	−3.00	1.17	−3.00
ILE	0.79	1.24	1.38	−3.00	−3.00	−3.00	−3.00	−3.00	−3.00
LEU	−3.00	−3.00	−3.00	2.10	−3.00	−3.00	1.27	2.25	2.36
LYS	−3.00	0.66	1.19	−3.00	−3.00	−3.00	−3.00	2.01	−3.00
MET	−3.00	−3.00	−3.00	1.41	−3.00	−3.00	−3.00	−3.00	−3.00
PHE	1.22	−3.00	1.82	−3.00	1.39	−3.00	−3.00	−3.00	−3.00
PRO	−3.00	−3.00	1.10	−3.00	−3.00	−3.00	1.85	−3.00	−3.00
SER	−3.00	0.89	−3.00	−3.00	−3.00	1.43	−3.00	−3.00	0.59
THR	−3.00	−3.00	−3.00	−3.00	−3.00	2.17	−3.00	−3.00	−3.00
TRP	1.53	−3.00	−3.00	−3.00	−3.00	−3.00	−3.00	−3.00	−3.00
TYR	2.09	−3.00	−3.00	−3.00	−3.00	−3.00	1.87	−3.00	−3.00
VAL	0.65	1.37	−3.00	−3.00	−3.00	−3.00	−3.00	−3.00	1.12

### Evaluation of MHC Class II Binding Prediction Performance

All three prediction approaches were evaluated on a common benchmark of MHC class II binding peptides to HLA-DRB*0101. The pair-potential method was applied using C_m_ interaction centers, and a distance cutoff of 7.5 Å. The molecular-dynamics based method used the scoring matrix in Table 1, and the contact map method used the scoring matrix in Table 2. For each peptide in the evaluation set, most of which are 15-mers, all possible 9-mer cores were evaluated, and the core with the highest affinity was chosen. The overall performance of the prediction methods was evaluated by their ability to distinguish binding peptides in the set with an IC50<1,000 nM from those with a weaker affinity, as evaluated by a ROC curve.

**Figure 4:**
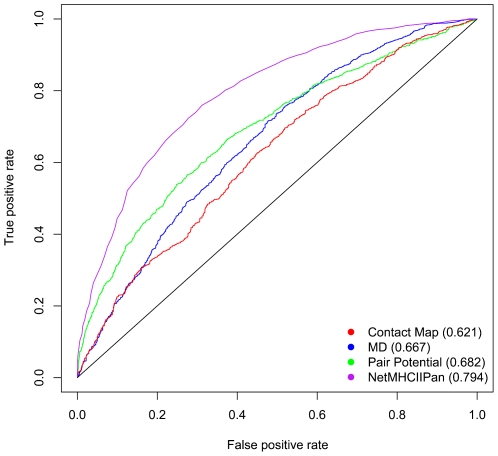
Performance of three *ab initio* structure based prediction methods and NetMHCIIpan using HLA DRB1*0101 as an example. The prediction results of three methods applied to HLA DRB1*0101 binding data are shown in the ROC plot. The ROC curves were generated by plotting the true positive rate (y-axis) against the false positive rate (x-axis). The AUC values for the three methods were shown in parentheses.

Figure 4 shows the ROC curves for the three methods. For the statistical pair potential method, the AUC value was 0.682±0.009, while it was 0.667±0.009 for the molecular dynamics method and 0.621±0.010 for the contact-map method. This is significantly better than a random value of AUC = 0.5 (p-value<0.00001 using standard error z-statistics). As a comparison, the ROC curve for the prediction method NetMHCIIpan was included in Figure 4 as well. As described previously [17] NetMHCIIpan utilizes measured peptide binding data from all MHC class II alleles, and can extrapolate predictions to new alleles for which no such data are available. The performance of this method (trained excluding all HLA-DRB1*0101 data) with an AUC value of 0.794 is substantially higher than all structure based predictions.

## Discussion

Predicting peptide binding for MHC class II molecules remains a challenging problem [27]. While available prediction methods showed success in peptide binding prediction, their performance is much worse than the performance of the methods for MHC class I binding prediction [2]. In addition, existing methods typically depend on large sets of experimentally measured binding affinities and are not applicable to MHC alleles that lack such data. The three approaches described here were developed independently with the goal of deriving peptide:MHC binding predictions that do not require any binding data. The resulting prediction performances are significantly better than random. However, they are still substantially lower than the best performing sequence based class II prediction methods available (AUC∼0.863) [27]. We have chosen to present our results together in order to support the notion that generating structure-based predictions of peptide:MHC binding without using binding data is unlikely to give satisfactory results.

While it is surely possible to improve on the approaches presented here, a large gap to satisfactory prediction qualities remains. This is especially true as the evaluations conducted here for the DRB1*0101 MHC molecules constitute a best case scenario, as this is the molecule with the largest amount of 3D structures information available. One straightforward approach to improve upon the prediction quality would be to make a consensus of the three methods applied here. Minimally, this would achieve the prediction performance of the convex hull of the ROC curves shown in Figure 4. Still, such a consensus would have an AUC value of less than 0.70, which is conventionally accepted as a usable prediction performance of sequence based prediction methods.

It has to be stressed that we are not claiming that the use of structural data has no place in peptide:MHC binding predictions. We are explicitly referring here to limitations of ‘*ab initio*’ methods, meaning those developed in the absence of any binding data. In fact, the use of structural methods in combination with binding data is promising, as shown by which peptide binding data is used to parameterize structure based scoring functions. Also, implicitly structural data are used in the ‘Pan’ approaches, which include representations of peptide contact residues in the MHC binding pocket molecule positions [12], [13], [15], [16], [17].

Another requirement we placed on the methods implemented here is that they needed to be capable of performing predictions on realistic sized datasets in a reasonable time frame. The standard application of these binding predictions is to scan sets of proteins or entire genomes for potential binding peptides. This easily leads to tens of thousands of predictions that have to be made, and rules out the use of very computationally expensive prediction approaches. For example, a prediction that would rely on generating molecular dynamics simulations for a peptide of interest is simply not practical. The cost of performing a peptide:MHC binding experiment, which is routinely feasible for less than $50, places a boundary on the amount of computation time that is justifiable in a real-world application. While generating the scoring matrix is a time consuming process for our MD based approach (performing a 4 ns MD simulation for a peptide:MHC complex takes about 2 weeks on a 64 nodes Linux cluster), our MD based prediction method could easily manage genome scale peptide binding prediction once the scoring matrices are generated. The predictive matrix of the contact map based approach only takes seconds to produce, and can easily handle genome scale predictions, similar to the MD based approach. In contrast, the pair potential method requires generating 3d models of peptide:MHC molecules for each possible register, which takes minutes per peptide, and makes genomic scale predictions more problematic.

The work presented here shows similar predictive performance as the early attempts to use protein structure and threading techniques to predict peptide binding to MHC molecules [3], [28]. Most structure-based MHC prediction algorithms are not available online on the web, making large scale benchmarking of their predictive performance impossible. Exceptions to this are PREDEP [4], and MHCPred [29] where online prediction servers are available covering a limited set of MHC molecules. In recent large-scale benchmark calculations both of these methods have been shown to under-perform significantly when compared to state-of-the-art data-driven methods [2], [30]. In particular, the MHCpred method was shown to achieve a predictive performance of 0.565 AUC when evaluated on a set of more than 1000 HLA-DRB1*0101 peptides [30], suggesting that this method does not outperform the methods considered in this work.

Experimental data had suggested that residues outside of the MHC class II binding groove contribute to binding [31], [32] and prediction methods have been developed incorporating such residues with considerable success [30] Our analysis of residue flexibility with the MD simulation data supports this notion. While peptide residues more than one amino acids away from the 9mer binding core are unlikely to contribute to binding due to excessive flexibility, the +1 and −1 residues could play detectable roles in binding as they share similar flexibility with other core residues. Our analysis of hydrogen bonds (data not shown) detected two stable hydrogen bonds formed between the +1 peptide residue and MHC residues and another two stable hydrogen bonds formed between the −1 peptide residue and MHC residues. We further analyzed the roles of those resides in binding by carrying out free energy calculation similar to the core residues. The resulting energy (data not shown) suggested that the +1 and −1 positions have small standard deviations similar to the non-anchor core positions 2, 3, 5 and 8. This suggested that their contribution to binding is mostly due to backbone interactions. Those results provided dynamic evidence supporting the roles of residues immediately outside of the binding groove in peptide:MHC interaction and suggested that predictive methods should incorporate residues outside of the binding core.

In summary, we have developed and tested three ‘*ab initio*’ structure based binding approaches that do not require peptide:MHC binding information, and found their prediction performance to be limited. We believe, it is nevertheless important to publicize this essentially negative finding as the approaches tested here have an obvious appeal and similar approaches are likely be pursued repeatedly. Also, we would like to be proven wrong, and will be convinced of the usefulness of *ab initio* structure based predictions by a method that is publicly available, capable of performing predictions for 1,000 peptides in less than a day, and was developed without requiring peptide:MHC binding data for a complex parameterization.

## Materials and Methods

The materials and methods section is separated into three parts corresponding to the three distinct approaches for MHC peptide binding.

### The Statistical Pair-Potential-Based Method

This method is based on deriving heuristic potentials between amino acids based on the frequency with which pairs of amino acids occur at a given distance in a large set of protein structures. These potentials are used to assign a heuristic binding affinity to homology modeled peptide:MHC binding complexes.

#### Statistical Pair-Potential

The statistical potential is defined as a logarithm of the ratio of the probability of observation against the probability of expectation. Here, we take the form adopted by Samudrala et al. [33] to calculate a potential from the count of observations:
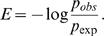
(1.1)To estimate the probability *P_obs_*, we counted the number of obser vations of amino acid pairs (*a*, *b*), within a distance *r* in a representative set of protein structures. To obtain *P_exp_* , we assume that for any given pair of amino acids (a, b), the distribution is homogenous for a given distance *r*, i.e., P(r|a,b) = P(r). The potential is hence calculated as:
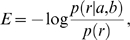
(1.2)where log is the the natural logarithm.

To predict peptide:MHC binding interactions, we are only interested in the inter-chain interactions between the peptide and the MHC molecule. To focus the potential on such non-local interactions, only amino acid pairs with a sequence separation greater than 9 amino acids were included when calculating the potential function.

The Culled PDB dataset from Wang and Dunbrack [34] was used to build the knowledge-based potential function. This collection of data is composed of 1202 high-resolution (resolution cutoff 2.0 Å) crystal structures of globular proteins with sequence length between 300 and 460 residues (MHC protein sequence size ±80 residues). The pair-potential was calculated using a distance bin of 0.25 Å. A penalty term was assigned to the potential function for distances closer than 1.0 Å to account for steric repulsion. The steric penalty was set to 2.0.

Finally, to limit the computational cost and to optimize the potential scoring function, only pairwise interactions up to a specific distance cutoff value were included [33], [35].

#### Reduced Models for Proteins

The calculation of the potential functions was based on a reduced model for protein structures where the pair-wise interactions of residues are represented by distance-dependent interactions between centroids. A number of schemes to represent the interaction centres of amino acid residues were tested: Cα, Cβ, Cm. In the Cα scheme, a residue is represented by its alpha carbon; in Cβ by the beta carbon in the sidechain (a virtual betacarbon is calculated for glysine); in Cm by the centre of mass of the heavy sidechain atoms (non-hydrogen). Other types of centroid definitions could be considered including backbone atoms of the residue. However, backbone conformations are highly conserved for different residue types and inclusion of such atoms in the centroid description would predominantly lead to residue-type specific shifts in centroid location towards the Cβ position. Furthermore, sidechain center of mass centroids have earlier been show to perform well for knowledge-based potential functions [36].

#### Homology Modelling

The models of peptide:MHC complexes were predicted using Modeller 8.v2 [37]. Modeller generates an ensemble of models using an initial random seed, and selects those with as little violation as possible to the spatial restrains derived from the alignment and expressed as probability density functions (PDFs). The PDFs restrain Cα-Cα and backbone N-O distances, as well as backbone and side-chain dihedral angles for different residue types. A pool of 42 templates was used to build peptide:MHC binding models (Table S5). For each peptide, three MHC complex models were constructed from the template pool using different initial seeds for Modeller. To obtain a predicted affinity for a given peptide, the three peptide:MHC models were evaluated in the pair-potential, and the final binding score was obtained as the simple average of the three binding scores.

#### Parameter estimation based on MHC-I data

To assess the performance of the three protein geometric representation models Cα, Cβ, and Cm and to estimate the optimal distance cutoff for pairwise interactions in the potential function, we performed benchmarks based on a large set of 37384 MHC class I binding data restricted to 42 MHC class I alleles used in the original NetMHCpan publication [16]. To obtain fair statistics covering different HLA molecules, we sampled randomly 100 data points from each of the 12 HLA class I supertypes. Furthermore, to fairly represent the diversity within a given supertype, an equal number of binding data were sampled from each allele within the supertype. This formed a representative dataset for the peptide:MHC binding data. This training set contains 1174 peptides with affinity data (the B39 supertype only had 74 binding measurements). The remaining peptide data were used to form the evaluation data set, which contains 36210 peptide:MHC binding data.

### The Molecular Dynamics-Based Method

This method is based on sampling the configurations that a peptide adopts in a molecular dynamics simulation of a peptide:MHC binding complex. Using *in silico* mutations of the peptides in each configuration, an average contribution to binding free energy of each possible amino acid in each position of the peptide core is assigned.

#### Molecular dynamics simulation

The molecular dynamics (MD) simulation was performed with the software package NAMD [38] using the CHARMM22 force field [39] with an explicit water model. The structure of the MHC class II molecule in complex with peptide epitope (PDB ID 2G9H) was taken from the Protein Data Bank [40]. The simulation was performed with the following protocol. The peptide:MHC complex was solvated in a box of TIP3 water with at least 10 Å distance between protein and the boundary of the water box. The system was first minimized with 10,000 steps of steepest descent followed by 100,000 steps of conjugate gradient descent. The MD simulation time step was 2 fs, and trajectory was saved every 1 ps. The particle mesh Ewald method was used to treat long-range electrostatic interactions and bond lengths involving hydrogen atoms were constrained with the SHAKE algorithm [41]. Constant temperature was controlled by Langevin dynamics, and pressure was maintained by using Nosé-Hoover Langevin piston pressure control. For the purpose of free energy calculation, 100 snapshots were taken from the last 1 ns of the 4 ns MD simulation trajectory.

#### In silico mutation of the peptide:MHC complex

For each of the 100 snapshot structures of the MD simulation, the following in silico mutations were performed. For each position of the 9-mer binding core of the peptide, 19 mutated structures were generated where each structure contained a mutation of the core residue to one of the other 19 amino acids. Thus, for each snapshot 171 (19×9) mutated structures were generated that covered all possible amino acids at each of the core position of the peptide. The mutations were generated with the “Mutate Residue” Plugin of the VMD software [42]. The mutated structures were minimized with 10,000 steps of conjugate gradient descent using NAMD before they were subjected to binding free energy calculation.

#### Calculating binding free energy contribution of core peptide residues

The contribution to binding free energy was calculated for all 20 amino acids at each position of the 9-mer binding core via a computational alanine scanning like approach:
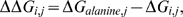
(2.1)where 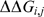 is the contribution to binding free energy of residue *i* at peptide core position *j*, 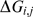 is the binding free energy between the MHC class II molecule and the peptide where the residue at position *j* was mutated to amino acid *i* and 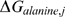 is the binding free energy between MHC class II molecule and the peptide where residue at position *j* is mutated to alanine.

**Figure 5:**
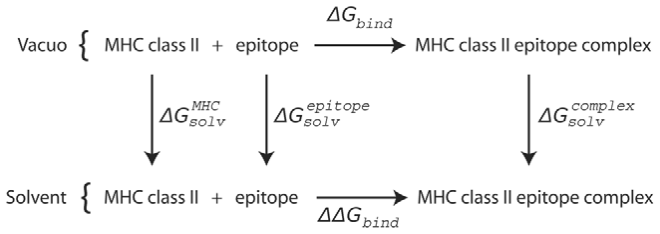
Thermodynamic cycle used to calculate the binding free energies between MHC class II molecule and the epitope peptide. The diagram shows the thermodynamic cycle for the binding of a MHC class II molecule and a epitope peptide, in both the solvated phase and *in vacuo*. The free energy of binding in solvent can be calculated by the following equation: 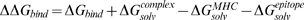.

The absolute binding free energy between the MHC class II molecule and peptide was calculated with the molecular mechanics-Poisson-Boltzmann surface area (MM-PBSA) approach according to the thermodynamic cycle shown in Figure 5. In this formulation, the binding free energy was the sum of gas phase contribution, 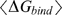, the desolvation energy upon binding, 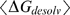, and an entropic term, -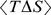:
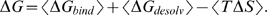
(2.2)The brackets, <>, denote an average over snapshots taken from the MD simulation trajectories.

The entropy term was omitted from our calculation since previous studies have shown that this term is canceled out when comparing systems with a single point mutation [43].

The gas-phase contribution to the binding free energy, 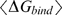, is the sum of the van der Waals and electrostatic interaction between MHC class II molecule and peptide and the difference in the internal energy between the peptide:MHC complex and the individual molecules of MHC class II and peptide. Those energies were calculated with the “NAMD Energy” plugin of VMD using the provided default parameters.

The solvation contribution for binding free energy, 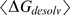, is the difference between the solvation energy of the peptide:MHC complex and those of the isolated MHC class II molecule and peptide. The solvation energy is divided into the electrostatic contribution and the non-polar contribution. The non-polar contribution to the solvation energy was calculated with an empirical formula: Δ*G_np,solv_* = σ×*SASA* where SASA is the solvent-accessible surface area and σ is a constant value of 0.0072 kcal/Å2 [19]. The electrostatic contribution to solvation energy was calculated by solving the Poisson- Boltzmann equation with Delphi [44] at 0.10 M salt. The partial charges and atomic radii were taken from the CHARMM22 force field. The interior of the molecular surface of the solute molecule (calculated with a 1.4 Å probe sphere) was assigned a dielectric constant of epsilon = 2, whereas the exterior aqueous phase was assigned a value of epsilon = 80. Debye–Hückel boundary conditions and five focusing steps were used with a cubic grid size of 155.

### The Contact Map-Based Method

The contact-based method implements a simple peptide:MHC contact model that assumes the following: (i) the peptide residues interact independently with the MHC molecule and (ii) the probability of an amino acid to be in a certain position of the peptide core is proportional to the average number of atomic contacts made by that amino acid in that position with the MHC molecule in 3D structures of peptides in complexes with MHC class II of a particular allele. The structures used for the method development are provided in Table 3.

**Table 3:** Structural data used throughout this study to derive the MHC class II structure-based binding predictions.

Allele	PDB ID	Resolution(A)	R-Value	R-free	peptide sequence	peptide core	peptide chain ID	MHC alpha chain ID	MHC beta-chain ID
DRB1*0101	2FSE	3.1	0.222	0.295	AGFKGEQGPKGEPG	FKGEQGPKG	E	A	B
DRB1*0101	1KLG	2.4	0.206	0.246	GELIGILNAAKVPAD	IGILNAAKV	C	A	B
DRB1*0101	1SJE	2.45	0.196	0.223	PEVIPMFSALSEGATP	VIPMFSALS	C	A	B
DRB1*0101	1AQD	2.45	0.216	0.279	GSDWRFLRGYHQYA	WRFLRGYHQ	C	A	B
DRB1*0101	1T5W	2.4	0.231	0.255	AAYSDQATPLLLSPR	YSDQATPLL	C	A	B
DRB1*0101	2G9H	2	0.215	0.252	PKYVKQNTLKLAT	YVKQNTLKL	C	A	B

The different columns give the MHC allele name, PDB identifier, resolution of X-ray structure, the R-free structure quality value, the peptide sequence, the peptide binding core as defined from the crystal structure, followed by the PDB chain ID for the peptide, MHC alpha, and MHC beta chains, respectively.

#### Constructing the MHC allele-specific PSSMs

The elements of a position-specific scoring matrix (PSSM) were calculated as follows:
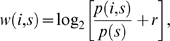
(3.1)where p(i,s) is the probability of the amino acid s at position i, and r = 0.05 is a small value added to avoid underflow when p(i,s) = 0.

When more than one structure for an allele was considered, the probabilities p(i,s) in the equation above were calculated as follows:
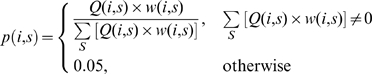
(3.2)where *Q(i,s)* is the number of times residue *s* is found at position *i* in all peptide core sequences in the analyzed structures.
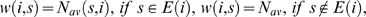
where *E(i)* is a set of amino acids at the position *i* in all core sequences from the analyzed structures; *N_av_(s,i)* is an average number of contacts that amino acid *s* at the position *i* makes with MHC; and *N_av_* is the average of contacts over all residues in all analyzed structures of peptide:MHC complexes for a particular allele.

If only one structure was considered for the allele, the probabilities *p(i,s)* were calculated using the following equation:
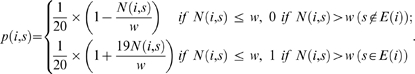
(3.3)where *N(s,i)* is a number of contacts that amino acid *s* at the position *i* of the core makes with the MHC molecule and *w* is a free parameter that was taken as equal to the average number of contacts per residue over all core residues.

### Benchmark Data Sets + Performance Evaluation Metrics

The evaluation of methods was performed using HLA DRB1*0101 binding data described in detail elsewhere [27]. Briefly, the dataset contains 3,882 experimentally measured peptide:MHC binding affinities. The binding affinities were expressed in terms of IC50 values and the experiments were all carried out as described before [45]. For evaluation purpose, the peptides were classified into 2939 binders (experimental IC50<1000 nM) and 943 non-binders (experimental IC50> = 1000 nM). The receiver operating characteristic (ROC) curves [46] were used to measure the performance of prediction algorithms. The ROC curve is generated by plotting the true positive rate against the false positive rate while changing the cutoff from the highest to lowest prediction score. The area under the ROC curve (AUC) can be used to measure prediction performance where 0.5 is random prediction and 1.0 is perfect prediction. The actual plotting of ROC curve and calculation of AUC were carried out with the ROCR [47] package of R [48]. Standard errors for AUC values were calculated according to [49] as:
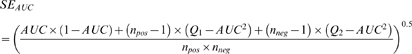
(4.1)Where AUC is the area under the curve, n_pos_ and n_neg_ are the number of positive and negative binding peptides in the test set respectively, and Q_1_ and Q_2_ are calculated as
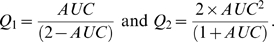



## Supporting Information

Table S1 Benchmark performance of methods. Columns are: name of allele, supertype, number of peptides, followed by the Pearsons correlation between the logarithm of the measured binding affinity and Modeller energy, pair potential energy based on C_α_, C_β_ and C_m_ centre of interaction, respectively. NN refers to the leave-one-out performance of NetMHCpan taken from Nielsen et al, 2007 [16]. The pair-potential cutoff values for C_α_, C_β_ and C_m_ were 20 Å, 20 Å, and 7.5 Å, respectively (see figure 1).
(0.08 MB DOC)

Table S2 Structures of peptide:MHC class II complexes used in the benchmarking contact-based method.
(0.04 MB DOC)

Table S3 The results of the peptide:MHC class II binding affinity prediction using the contact-based method.
(0.06 MB DOC)

Table S4 Number of atomic contacts for peptide core residues in complexes with HLA-DRB1*0101, counting hydrogen bonds, van der Waals, and hydrophobic interactions.
(0.04 MB DOC)

Table S5 PDB templates used in homology modeling of the structures of peptide:MHC-I complexes for the pair potential method.
(0.03 MB DOC)
