# Supplementary material for: Limitations of Ab Initio Predictions of Peptide Binding to MHC Class II Molecules
Source: PLoS One. 2010 Feb 17;5(2):e9272. doi: 10.1371/journal.pone.0009272 (PMC2822856; doi:10.1371/journal.pone.0009272)
Supplement: Table S1 — Benchmark performance of methods. Columns are: name of allele, supertype, number of peptides, followed by the Pearsons correlation between the logarithm of the measured binding affinity and Modeller energy, pair potential energy based on Cα, Cβ and Cm centre of interaction, respectively. NN refers to the leave-one-out performance of NetMHCpan taken from Nielsen et al, 2007 [16]. The pair-potential cutoff values for Cα, Cβ and Cm were 20 Å, 20 Å, and 7.5 Å, respectively (see figure 1). (0.08 MB DOC) [file pone.0009272.s001.doc]

**Table S1: Benchmark performance of methods.** Columns are: name of allele, supertype, number of peptides, followed by the Pearsons correlation between the logarithm of the measured binding affinity and Modeller energy, pair potential energy based on C_α_, C_β_ and C_m_ centre of interaction, respectively. NN refers to the leave-one-out performance of *NetMHCpan* taken from Nielsen et al, 2007 [16]. The pair-potential cutoff values for C_α_, C_β_ and C_m_ were 20 Å, 20 Å, and 7.5 Å, respectively (see figure 1).

| Allele | ST | # | Modeller | C_α_ | C_β_ | C_m_ | NN |
| --- | --- | --- | --- | --- | --- | --- | --- |
| A0101 | A1 | 1163 | 0.11 | 0.09 | 0.11 | 0.30 | 0.46 |
| A0201 | A2 | 3866 | 0.10 | 0.55 | 0.55 | 0.51 | 0.87 |
| A0202 | A2 | 1437 | 0.14 | 0.36 | 0.37 | 0.37 | 0.81 |
| A0203 | A2 | 2036 | -0.04 | 0.46 | 0.46 | 0.38 | 0.87 |
| A0206 | A2 | 2045 | 0.04 | 0.46 | 0.47 | 0.38 | 0.79 |
| A0211 | A2 | 131 | 0.03 | 0.14 | 0.10 | 0.02 | 0.65 |
| A0212 | A2 | 103 | 0.03 | 0.44 | 0.35 | 0.40 | 0.88 |
| A0216 | A2 | 47 | 0.02 | 0.09 | 0.08 | 0.00 | 0.76 |
| A0219 | A2 | 127 | -0.04 | 0.20 | 0.18 | 0.32 | 0.74 |
| A0301 | A3 | 2472 | 0.11 | 0.03 | 0.02 | 0.09 | 0.79 |
| A1101 | A3 | 2231 | -0.20 | -0.04 | -0.06 | -0.01 | 0.84 |
| A2301 | A24 | 134 | 0.32 | 0.26 | 0.29 | 0.33 | 0.77 |
| A2402 | A24 | 385 | -0.11 | -0.35 | -0.39 | 0.35 | 0.81 |
| A2403 | A24 | 288 | 0.29 | 0.35 | 0.39 | 0.42 | 0.82 |
| A2601 | A26 | 999 | 0.07 | 0.18 | 0.23 | 0.16 | 0.69 |
| A2602 | A26 | 43 | -0.16 | -0.04 | -0.10 | 0.26 | 0.52 |
| A2902 | A26 | 127 | 0.23 | 0.33 | 0.41 | 0.57 | 0.68 |
| A3001 | A3 | 915 | 0.05 | -0.11 | -0.13 | 0.02 | 0.69 |
| A3002 | A1 | 42 | 0.13 | 0.10 | 0.20 | 0.19 | 0.79 |
| A3101 | A3 | 2107 | -0.04 | -0.03 | -0.06 | 0.16 | 0.77 |
| A3301 | A3 | 1124 | 0.03 | 0.02 | -0.01 | 0.08 | 0.65 |
| A6801 | A3 | 1125 | -0.03 | -0.05 | -0.07 | -0.11 | 0.62 |
| A6802 | A2 | 1424 | 0.02 | 0.31 | 0.34 | 0.05 | 0.74 |
| A6901 | A2 | 1638 | -0.06 | 0.35 | 0.35 | 0.26 | 0.76 |
| B0702 | B7 | 1552 | -0.18 | 0.04 | 0.05 | 0.12 | 0.56 |
| B0801 | B8 | 779 | 0.11 | 0.03 | 0.02 | 0.16 | 0.62 |
| B0802 | B8 | 691 | 0.33 | -0.05 | -0.07 | 0.10 | 0.59 |
| B1501 | B62 | 1184 | 0.18 | 0.22 | 0.27 | 0.37 | 0.40 |
| B1801 | B8 | 257 | 0.00 | 0.14 | 0.25 | 0.33 | 0.76 |
| B2705 | B27 | 1157 | 0.20 | -0.13 | -0.09 | 0.28 | 0.06 |
| B3501 | B7 | 962 | -0.11 | 0.21 | 0.26 | 0.34 | 0.69 |
| B4001 | B44 | 1237 | 0.05 | -0.05 | -0.01 | 0.09 | 0.58 |
| B4002 | B44 | 98 | 0.13 | -0.14 | -0.08 | 0.06 | 0.82 |
| B4402 | B44 | 99 | 0.29 | -0.29 | -0.19 | 0.04 | 0.79 |
| B4403 | B44 | 99 | 0.14 | -0.32 | -0.26 | -0.07 | 0.76 |
| B4501 | B44 | 94 | -0.28 | -0.29 | -0.24 | -0.10 | 0.56 |
| B5101 | B7 | 224 | -0.04 | 0.26 | 0.27 | 0.32 | 0.58 |
| B5301 | B7 | 234 | -0.02 | 0.12 | 0.14 | 0.39 | 0.74 |
| B5401 | B7 | 235 | -0.10 | 0.28 | 0.31 | 0.16 | 0.57 |
| B5701 | B58 | 9 | 0.00 | 0.22 | 0.22 | 0.07 | 0.38 |
| B5801 | B58 | 1290 | -0.09 | 0.21 | 0.27 | 0.37 | 0.45 |
| Ave |  | 36210 | 0.04 | 0.11 | 0.13 | 0.21 | 0.67 |
