# Supplementary material for: Limitations of Ab Initio Predictions of Peptide Binding to MHC Class II Molecules
Source: PLoS One. 2010 Feb 17;5(2):e9272. doi: 10.1371/journal.pone.0009272 (PMC2822856; doi:10.1371/journal.pone.0009272)
Supplement: Table S2 — Structures of peptide:MHC class II complexes used in the benchmarking contact-based method. (0.04 MB DOC) [file pone.0009272.s002.doc]

**Table S2:** Structures of peptide:MHC class II complexes used in the benchmarking contact-based method.

| Allele | PDB ID | Resolution | R-Value | R-free | Peptide sequence | Peptide core | Peptide chain ID | MHC alpha chain ID | MHC beta-chain ID |
| --- | --- | --- | --- | --- | --- | --- | --- | --- | --- |
| DRB1*0101 | 2FSE | 3.1 | 0.222 | 0.295 | AGFKGEQGPKGEPG | FKGEQGPKG | E | A | B |
| DRB1*0101 | 1KLG | 2.4 | 0.206 | 0.246 | GELIGILNAAKVPAD | IGILNAAKV | C | A | B |
| DRB1*0101 | 1SJE | 2.45 | 0.196 | 0.223 | PEVIPMFSALSEGATP | VIPMFSALS | C | A | B |
| DRB1*0101 | 1AQD | 2.45 | 0.216 | 0.279 | GSDWRFLRGYHQYA | WRFLRGYHQ | C | A | B |
| DRB1*0101 | 1T5W | 2.4 | 0.231 | 0.255 | AAYSDQATPLLLSPR | YSDQATPLL | C | A | B |
| DRB1*0101 | 2G9H | 2 | 0.215 | 0.252 | PKYVKQNTLKLAT | YVKQNTLKL | C | A | B |
| DRB1*0301 | 1A6A | 2.75 | 0.246 | 0.325 | PVSKMRMATPLLMQA | MRMATPLLM | C | A | B |
| DRB1*0401 | 2SEB | 2.5 | 0.229 | 0.28 | AYMRADAAAGGA | MRADAAAGG | E | A | B |
| DRB1*0401 | 1J8H | 2.4 | 0.211 | 0.246 | PKYVKQNTLKLAT | YVKQNTLKL | C | A | B |
| DRB1*1501 | 1BX2 | 2.6 | 0.238 | 0.266 | ENPVVHFFKNIVTPR | VHFFKNIVT | C | A | B |
| DRB5*0101 | 1FV1 | 1.9 | 0.233 | 0.267 | NPVVHFFKNIVTPRTPPPSQ | FKNIVTPRT | C | A | B |
| DRB5*0101 | 1H15 | 3.1 | 0.256 | 0.31 | GGVYHFVKKHVHES | YHFVKKHVH | C | A | B |
| H-2-IAb | 1LNU | 2.5 | 0.211 | 0.245 | FEAQKAKANKAVDGGGG | AQKAKANKA | B: 1-13 | A | B |
| H-2-IAb | 1MUJ | 2.15 | 0.22 | 0.246 | PVSKMRMATPLLMQA | MRMATPLLM | C | A | B |
