# Supplementary material for: Limitations of Ab Initio Predictions of Peptide Binding to MHC Class II Molecules
Source: PLoS One. 2010 Feb 17;5(2):e9272. doi: 10.1371/journal.pone.0009272 (PMC2822856; doi:10.1371/journal.pone.0009272)
Supplement: Table S3 — The results of the peptide:MHC class II binding affinity prediction using the contact-based method. (0.06 MB DOC) [file pone.0009272.s003.doc]

**Table S3**. The results of the peptide:MHC class II binding affinity prediction using the contact-based method.

| **Allele** | **Structures Used, PDB ID** | **AUC Value for the interaction schema** | | | |
| --- | --- | --- | --- | --- | --- |
|  |  | **Hydrogen bonds, van der Waals, and hydrophobic interactions** | **4 Å contacts** | **Hydrogen bonds** | **Hydrogen bonds and van der Waals interactions** |
| **HLA-DRB1*0101** | 1KLG | **0.641** | 0.617 | *0.612* | 0.632 |
|  | 1SJE | 0.591 | **0.597** | 0.606 | *0.591* |
|  | 1AQD | **0.603** | *0.563* | 0.570 | 0.566 |
|  | 1T5W | **0.582** | 0.561 | 0.576 | *0.559* |
|  | 2FSE | **0.521** | 0.489 | *0.444* | 0.489 |
|  | 2G9H | **0.589** | 0.576 | *0.554* | 0.584 |
|  | 1KLG, 1SJE | **0.650** | 0.643 | *0.642* | 0.642 |
|  | 1KLG, 1SJE, 2G9H | **0.652** | 0.645 | *0.621* | 0.645 |
|  | 1AQD, 1KLG, 1SJE, 1T5W | **0.649** | 0.642 | 0.653 | *0.641* |
|  | 1AQD, 1KLG, 1SJE, 1T5W, 2G9H | **0.639** | 0.632 | *0.624* | 0.633 |
|  | 1AQD, 1KLG, 1SJE, 1T5W, 2FSE, 2G9H | **0.621** | 0.610 | *0.602* | 0.613 |
| **HLA-DRB1*0301** | 1A6A | 0.573 | 0.573 | *0.552* | **0.574** |
| **HLA-DRB1*0401** | 1J8H | **0.551** | 0.535 | *0.522* | 0.529 |
|  | 2SEB | **0.529** | 0.524 | *0.504* | 0.519 |
|  | 1J8H, 2SEB | 0.531 | **0.533** | *0.501* | 0.530 |
| **HLA-DRB1*1501** | 1BX2 | 0.554 | **0.563** | *0.541* | 0.557 |
| **HLA-DRB5*0101** | 1FV1 | 0.619 | **0.621** | 0.607 | *0.596* |
|  | 1H15 | **0.662** | 0.643 | 0.589 | *0.642* |
|  | 1FV1, 1H15 | 0.591 | *0.560* | 0.574 | **0.595** |
| **H-2-IAb** | 1LNU | **0.625** | 0.602 | *0.554* | 0.604 |
|  | 1MUJ | **0.561** | 0.558 | *0.514* | 0.556 |
|  | 1LNU, 1MUJ | 0.611 | **0.620** | *0.547* | 0.620 |
