# Supplementary material for: Limitations of Ab Initio Predictions of Peptide Binding to MHC Class II Molecules
Source: PLoS One. 2010 Feb 17;5(2):e9272. doi: 10.1371/journal.pone.0009272 (PMC2822856; doi:10.1371/journal.pone.0009272)
Supplement: Table S4 — Number of atomic contacts for peptide core residues in complexes with HLA-DRB1*0101, counting hydrogen bonds, van der Waals, and hydrophobic interactions. (0.04 MB DOC) [file pone.0009272.s004.doc]

**Table S4.** Number of atomic contacts for peptide core residues in complexes with HLA-DRB1*0101, counting hydrogen bonds, van der Waals, and hydrophobic interactions.

| **PDB ID** | **1AQD** | | **1KLG** | | **1SJE** | | **1T5W** | | **2FSE** | | **2G9H** | |
| --- | --- | --- | --- | --- | --- | --- | --- | --- | --- | --- | --- | --- |
| **Core Position** | **aa** | **Contacts Number** | **aa** | **Contacts Number** | **aa** | **Contacts Number** | **aa** | **Contacts Number** | **aa** | **Contacts Number** | **aa** | **Contacts Number** |
| **1** | W | 121 | I | 57 | V | 49 | Y | 97 | F | 88 | Y | 116 |
| **2** | R | 48 | G | 19 | I | 27 | S | 19 | K | 15 | V | 31 |
| **3** | F | 62 | I | 40 | P | 30 | D | 25 | G | 13 | K | 33 |
| **4** | L | 79 | L | 73 | M | 76 | Q | 53 | E | 43 | Q | 50 |
| **5** | R | 30 | N | 8 | F | 15 | A | 10 | Q | 8 | N | 5 |
| **6** | G | 11 | A | 31 | S | 29 | T | 25 | G | 8 | T | 36 |
| **7** | Y | 88 | A | 25 | A | 26 | P | 37 | P | 49 | L | 48 |
| **8** | H | 21 | K | 18 | L | 35 | L | 27 | K | 20 | K | 11 |
| **9** | Q | 48 | V | 43 | S | 25 | L | 81 | G | 19 | L | 70 |
