# Supplementary material for: Limitations of Ab Initio Predictions of Peptide Binding to MHC Class II Molecules
Source: PLoS One. 2010 Feb 17;5(2):e9272. doi: 10.1371/journal.pone.0009272 (PMC2822856; doi:10.1371/journal.pone.0009272)
Supplement: Table S5 — PDB templates used in homology modeling of the structures of peptide:MHC-I complexes for the pair potential method. (0.03 MB DOC) [file pone.0009272.s005.doc]

**Table S5.** PDB templates used in homology modeling of the structures of peptide:MHC-I complexes for the pair potential method.

| MHC Allele | Number of templates | PDB ID |
| --- | --- | --- |
| A0201 | 23 | 1DUZ, 1B0G, 1EEY, 1EEZ, 1ILY, 1I7R, 1I7T, 1I7U, 1QEW, 1QR1, 1S8D, 1S9W, 1S9X, 1S9Y, 1T1W, 1T1X, 1T1Y, 1T1Z, 1T20, 1T21, 1T22, 1TVB, 1TVH |
| A0101 | 1 | 1W72 |
| A1101 | 2 | 1Q94, 1X7Q |
| B0801 | 1 | 1MI5 |
| B1501 | 2 | 1XR9, 1XR8 |
| B2705 | 6 | 1JGE, 1UXS, 2BST, 1OGT, 1W0V, 2BSR |
| B3501 | 1 | 1CG9 |
| B4402 | 1 | 1M6O |
| B4403 | 2 | 1N2R, 1SYS |
| B5101 | 1 | 1E27 |
| B5301 | 2 | 1A1M, 1A1O |
|  | 42 |  |
